# Bevacizumab increases the risk of anastomosis site leakage in metastatic colorectal cancer

**DOI:** 10.3389/fonc.2022.1018458

**Published:** 2022-10-21

**Authors:** Seijong Kim, Jung Kyong Shin, Yoonah Park, Jung Wook Huh, Hee Cheol Kim, Seong Hyeon Yun, Woo Yong Lee, Yong Beom Cho

**Affiliations:** ^1^ Department of Surgery, Samsung Medical Center, Sungkyunkwan University School of Medicine, Seoul, South Korea; ^2^ Department of Health Sciences and Technology, SAIHST, Sungkyunkwan University, Seoul, South Korea; ^3^ Department of Biopharmaceutical Convergence, Sungkyunkwan University, Seoul, South Korea

**Keywords:** colorectal (colon) cancer, bevacizumab, stage IV, anastomotic leak in colorectal surgery, chemotherapy

## Abstract

**Background:**

Bevacizumab is a humanized monoclonal antibody against vascular endothelial growth factor and is used in combination with first-line chemotherapy in the treatment of metastatic colorectal cancer. One of the side effects of bevacizumab is gastrointestinal perforation. This study was designed to identify the effect of bevacizumab in intestinal anastomosis site healing.

**Methods:**

From January 2010 to December 2020, patients diagnosed with stage IV colorectal cancer treated with palliative chemotherapy or chemoradiotherapy followed by radical surgery were retrospectively reviewed. Clinical signs or symptoms and computed tomography were tools used for diagnosing anastomosis site leakage. The patients were divided into two groups, the bevacizumab group (n = 136) and the non-bevacizumab group (n = 124).

**Results:**

Among the 260 patients 14 (5.4%) patients were diagnosed with anastomosis site leakage. In the bevacizumab group, 13 (9.6%) patients were diagnosed with anastomotic leakage. In the non-bevacizumab group, 1 (0.8%) patient was diagnosed with anastomotic leakage. Anastomosis site leakage was significantly higher in the bevacizumab treatment group (P < 0.001). In the bevacizumab group, period of drug discontinuation before surgery was factor associated with anastomosis site leakage in multivariable analysis (P = 0.031).

**Conclusion:**

Stage IV colorectal patients treated with bevacizumab before radical surgery for primary cancer should be carefully observed of anastomosis site leakage after surgery, and the period of drug discontinuation before surgery should be longer than 5 weeks to avoid anastomosis site leakage.

## Introduction

Bevacizumab (Avastin^®^) is a humanized monoclonal antibody against vascular endothelial growth factor (VEGF) used to inhibit VEGF function and, as a result, inhibit tumor angiogenesis (1). Fluoropyrimidine-based chemotherapy combined with bevacizumab in the first and second-line treatments of metastatic colorectal cancer significantly increased oncologic outcomes in several randomized controlled trials (2, 3). However, the antiangiogenic effect of bevacizumab inhibits the capillary beds of the small bowel villi, contributing to gastrointestinal perforation by provoking the regression of normal blood vessels in the gastrointestinal tract (4). Several studies showed an increased risk of gastrointestinal perforations in patients treated with bevacizumab (5, 6). In an animal model, the administration of bevacizumab inhibited angiogenesis in the intestinal anastomosis site, resulting in a decrease in a-SMA accumulation and collagen deposition in bowel anastomosis site tissue, which might affect the healing of intestinal anastomosis (7). Because of the antiangiogenic effect of bevacizumab, the discontinuation of bevacizumab is recommended at least 6 weeks before surgery (8). Therefore, this study was conducted to evaluate the effect of bevacizumab on intestinal anastomosis site healing in stage IV colorectal cancer patients who underwent preoperative chemotherapy.

## Methods

**Figure 1:**
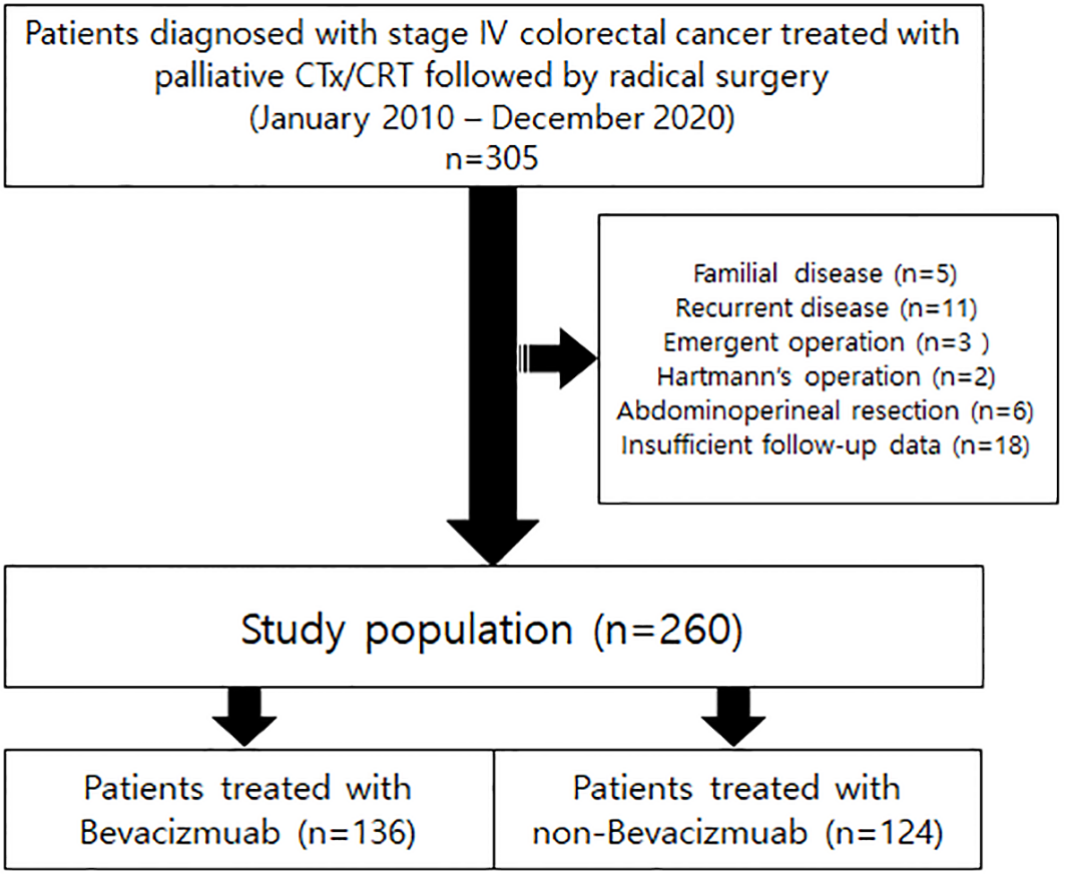
Flowchart showing study population selection.

From January 2010 to December 2020, patients diagnosed with stage IV colorectal cancer treated with palliative chemotherapy or chemoradiotherapy followed by radical surgery were retrospectively reviewed. Patients with familial disease, recurrent disease, emergent operations, or who underwent abdominoperineal resections, which has no anastomosis site, or without appropriate follow-up data were excluded from the cohort. A flowchart of patient selection is illustrated in **Figure 1**. A total of 260 patients were enrolled. This study was reviewed and approved by the Institutional Review Board of Samsung Medical Center (No. 2021-10-041).

Chemotherapy regimens were based on the National Comprehensive Cancer Network (NCCN) guidelines. In all the patients, chemotherapy with FOLFIRI/FOLFOX/XELOX was initiated with or without cetuximab/bevacizumab. All patients underwent tests of tumor gene status for KRAS/NRAS as well as MSI/MMR status. Patients with KRAS or NRAS mutation were not treated with cetuximab or panitumumab and treated with FOLFIRI/FOLFOX/XELOX alone or in combination with bevacizumab. When the disease progressed despite of first-line FOLFOX/XELOX based chemotherapy, chemotherapy regimen was altered. In previous oxaliplatin-based therapy without irinotecan, irinotecan ± aflibercept or pembrolizumab based chemotherapy was continued (8). Also, patients with low to mid-rectal cancer were discussed in the multidisciplinary meeting whether to undergo neoadjuvant radiotherapy before surgery.

Anastomosis site leakage was defined as a defect in the intestinal wall integrity at the colorectal or colo-anal anastomosis site, leading to a communication between the intraluminal and extraluminal compartments. An abscess in the pelvic cavity close to the anastomosis site was considered anastomotic leakage. Clinical symptoms and signs such as fever, tachycardia, abdominal pain or distension, leukocytosis, and elevated C-reactive protein (CRP) levels were indicators suspicious of anastomosis site leakage, so patients with the symptoms or signs listed above underwent computed tomography (CT). Peri-anastomotic loculated fluid containing air or anastomosis wall defects in contrast CT was considered anastomosis site leakage (9).

Statistical analyses were performed using Rex (Version 3.0.3, RexSoft Inc., Seoul, Korea) and SPSS version 27 (SPSS Inc., Chicago, IL, USA). The categorical variables were analyzed using the Chi-squared test, and the continuous variables were analyzed using the Mann-Whitney U-test. The logistic regression model was used to analyze the variables that could independently influence anastomosis site leakage. Variables with a P-value of < 0.1 in univariable analysis were entered into a multivariable analysis. A P-value of < 0.05 in the multivariable analysis was considered statistically significant.

## Results

Among the 260 patients, a total of 136 (52.3%) patients were treated with XELOX/FOLFOX/FOLFIRI combined with bevacizumab, 85 (32.7%) patients were treated with FOLFOX/FOLFIRI combined with cetuximab, 25 (9.6%) patients with XELOX only, 8 (3.2%) patients with FOLFOX only, 2 (0.8%) patients with FOLFIRI only, 2 (0.8%) patients with XELOX and XELIRI, 1 (0.3%) patient with FOLFIRI with aflibercept, and 1 (0.3%) patient with pembrolizumab. Three patients received 25Gy to 44Gy of radiation. The patients received a median of 6 courses of bevacizumab (minimum 3, maximum 42), and the median interval days between the last bevacizumab treatment to surgery was 43 days (range 16 – 240 days).

A comparison of the baseline clinicopathologic features between the groups is summarized in **Table 1**. Age, perineural invasion, and anastomosis site leakage were significantly different between the two groups (P = 0.012 P = 0.040, P < 0.001). In the group treated with bevacizumab, anastomosis site leakage was higher in patients with rectal cancer, and drug discontinuation periods shorter than 35 days (P = 0.020, P = 0.027; **Table 2**).

**Table 1:** Baseline clinicopathologic features of patients in the bevacizumab and non-bevacizumab groups.

	Non-bevacizumab (n=124)	Bevacizumab (n=136)	P-value
Age, Median (range)	54 (26–79)	57 (23–82)	0.005
Sex			0.250
Male	78 (62.9%)	76 (55.9%)	
Female	46 (37.1%)	60 (44.1%)	
BMI (kg/m^2^), Median (range)	23.9 (17.8-30.5)	24.0 (15.2-32.8)	0.889
Underlying DM			0.309
No	104 (83.9%)	120 (88.2%)	
Yes	20 (16.1%)	16 (11.8%)	
ASA score			0.549
1-2	114 (91.9%)	111 (89.0%)	
3	10 (8.1%)	15 (11.0%)	
Preoperative CEA			0.063
<5	89 (71.8%)	83 (61.0%)	
≥5	35 (28.2%)	53 (39.0%)	
Location			0.174
Colon	57 (46.0%)	74 (54.4%)	
Rectum	67 (54.0%)	62 (45.6%)	
Route of access			0.109
Open	53 (42.7%)	44 (32.4%)	
MIS	71 (57.3%)	92 (67.6%)	
Diverting stoma			0.065
No	98 (79.0%)	120 (88.2%)	
Yes	26 (21.0%)	16 (11.8%)	
Cancer obstruction			0.736
No	97 (78.2%)	104 (76.5%)	
Yes	27 (21.8%)	32 (23.5%)	
Cancer perforation			0.251
No	122 (98.4%)	134 (98.5%)	
Yes	2 (1.6%)	2 (1.5%)	
EBL (ml), Median (range)	243.5 (50-3000)	200 (50-2000)	0.064
Transfusion			0.231
No	112 (90.3%)	128 (94.1%)	
Yes	12 (9.7%)	8 (5.9%)	
Operation time (min)	283 (89-713)	281 (65-604)	0.329
Ulceration			0.196
No	33 (26.6%)	27 (19.9%)	
Yes	91 (73.4%)	109 (80.1%)	
Tumor size (cm)	4.0 (0-10.0)	3.9 (0-13)	0.527
Differentiation			0.668
Well to moderate	114 (91.9%)	123 (90.4%)	
Poor, SRC, MAC	10 (8.1%)	13 (9.6%)	
T stage			0.509
T0-T2	21 (16.9%)	18 (13.2%)	
T3-T4	103 (83.1%)	118 (86.8%)	
N stage			0.125
N-	31 (25.0%)	46 (33.8%)	
N+	93 (75.0%)	90 (66.2%)	
Circumferential resection margin			0.651
Negative	116 (93.6%)	130 (95.6%)	
Positive	8 (6.4%)	6 (4.4%)	
Harvested LN (n)			0.792
<12	15 (12.1%)	19 (14.0%)	
≥12	109 (87.9%)	117 (86.0%)	
Lymphovascular invasion			0.826
No	53 (42.8%)	61 (44.9%)	
Yes	67 (54.0%)	69 (50.7%)	
Undescribed	4 (3.2%)	6 (4.4%)	
Perineural invasion			0.040
No	27 (21.8%)	44 (32.4%)	
Yes	78 (62.9%)	82 (60.3%)	
Undescribed	19 (15.3%)	10 (7.3%)	
Tumor budding			0.953
No	62 (50.0%)	69 (50.7%)	
Yes	57 (46.0%)	61 (44.9%)	
Undescribed	5 (4.0%)	6 (4.4%)	
Anastomotic leakage			<0.001
No	123 (99.2%)	123 (90.4%)	
Yes	1 (0.8%)	13 (9.6%)	

BMI, Body mass index; CEA, Carcinoembryonic antigen; MIS, Minimally invasive surgery; EBL, Estimated blood loss; SRC, Signet ring cell; MAC, Mucinous adenocarcinoma; LN, Lymph node.

*The race of patients included in this study are all Asian.

**Table 2:** Baseline clinicopathologic features of patients with anastomotic leakage and non-leakage treated with bevacizumab.

	Without leakage (n=123)	With leakage (n=13)	P-value
Age	59 (24-82)	53 (23-69)	0.089
Sex			0.876
Male	69 (56.1%)	7 (53.8%)	
Female	54 (43.9%)	6 (46.2%)	
BMI	23.8 (15.2-32.8)	24.3 (20.0-29.1)	0.679
Underlying disease			
No	60 (48.8%)	7 (53.8%)	0.956
Yes	63 (51.2%)	6 (46.2%)	
Underlying DM			0.651
No	109 (86.2%)	11 (84.6%)	
Yes	14 (13.8%)	2 (15.4%)	
ASA score			0.686
1-2	109 (88.6%)	12 (92.3%)	
3	14 (11.4%)	1 (7.7%)	
Preoperative CEA			0.795
<5	76 (61.8%)	7 (53.8%)	
≥5	47 (38.2%)	6 (46.2%)	
Location			0.020
Colon	70 (56.9%)	3 (30.8%)	
Rectum	53 (43.1%)	10 (69.2%)	
Route of access			0.117
Open	37 (30.1%)	7 (53.8%)	
MIS	86 (69.9%)	6 (46.2%)	
Diverting stoma			0.651
No	109 (88.6%)	11 (84.6%)	
Yes	14 (11.4%)	2 (15.4%)	
Cancer obstruction			0.732
No	93 (75.6%)	11 (84.6%)	
Yes	30 (24.4%)	2 (15.4%)	
Cancer perforation			0.643
No	121 (98.4%)	13 (100%)	
Yes	2 (1.6%)	0 (0%)	
EBL (ml)	200 (5-2000)	150 (30-700)	0.631
Transfusion			0.562
No	116 (94.3%)	12 (92.3%)	
Yes	7 (5.7%)	1 (7.7%)	
Operation time (min)	282 (65-604)	273 (81-560)	0.441
Ulceration			0.722
No	24 (19.5%)	3 (23.1%)	
Yes	99 (80.5%)	10 (76.9%)	
Tumor size (cm)	4 (0-13)	3.5 (0-8.8)	0.900
Differentiation			0.360
Well to moderate	112 (91.1%)	11 (84.6%)	
Poor, SRC, MAC	11 (8.9%)	2 (15.4%)	
T stage			0.683
T0-T2	16 (13.0%)	2 (15.4%)	
T3-T4	107 (87.0%)	11 (84.6%)	
N stage			0.807
N-	42 (34.2%)	4 (30.8%)	
N+	81 (65.8%)	9 (69.2%)	
Circumferential resection margin			0.459
Negative	118 (95.9%)	12 (92.3%)	
Positive	5 (4.1%)	1 (7.7%)	
Harvested LN			0.492
<12	18 (14.6%)	1 (7.7%)	
≥12	105 (85.4%)	12 (92.3%)	
Lymphovascular invasion			0.570
No	55 (44.7%)	6 (46.2%)	
Yes	63 (51.2%)	6 (46.2%)	
Undescribed	5 (4.1%)	1 (7.6%)	
Perineural invasion			0.145
No	38 (30.9%)	6 (46.2%)	
Yes	77 (62.6%)	5 (38.5%)	
Undescribed	8 (6.5%)	2 (15.3%)	
Tumor budding			0.669
No	63 (51.2%)	6 (46.2%)	
Yes	55 (44.7%)	6 (46.2%)	
Undescribed	5 (4.1%)	1 (7.6%)	
Number of doses	6 (1-39)	7 (2-42)	0.482
Drug holiday			0.027
<35 days	22 (17.9%)	6 (46.2%)	
≥35 days	101 (82.1%)	7 (53.8%)	

BMI, Body mass index; CEA, Carcinoembryonic antigen; MIS, Minimally invasive surgery; EBL, Estimated blood loss; SRC, Signet ring cell; MAC, Mucinous adenocarcinoma; LN, Lymph node.

Among the all patients, 213 patients underwent radical operation of colorectal lesion with metastasectomy. The most common operation site for metastatic organ was liver. One hundred seventy-one (65.8%) patients underwent liver resection or intraoperative radio frequency ablation. Hemihepatectomy, sectionectomy, segmentectomy and wedge resection were conducted for the liver resection. The next common operation for metastasis was distant metastatic lymph node dissection (15.4%). Hysterectomy or oophorectomy were performed in 6.2% patients. Pneumonectomy, small bowel resection, operation for bladder/ureter were performed in 2.3% patients respectively. Splenectomy, pancreatectomy and wedge resection of stomach were performed in each one patient.

A total of 14 (5.4%) patients were diagnosed with anastomosis site leakage. The anastomosis site leakage events are described in detail in **Table 3**. Thirteen (92.85%) patients were treated with bevacizumab and 1 (7.15%) patient was treated with cetuximab. Among 14 patients, 1 (7.2%) was diagnosed before 7 postoperative days (PODs), 10 (71.4%) patients between 1 – 2 weeks, 3 (21.4%) patients between 3 – 4 weeks. 10 (71.4%) patients were diagnosed at hospitalization and 4 (28.6%) were diagnosed after discharge. 9 (64.3%) patients had body temperatures higher than 38 °C and 10 (71.4%) patients had elevated white blood cell (WBC) counts with elevated absolute neutrophil counts, and all patients had elevated CRP levels. Two (14.3%) patients had stable vital signs with elevated CRP levels. Among 9 patients with fever, 2 patients had septic shock and 1 patient had sepsis before emergent surgery.

**Table 3:** Detailed information and treatment of patients diagnosed with anastomosis site leakage.

No.	Sex	Age	Chemotherapy agent	No. of target agent doses	Drug discontinuation day/ Diagnosed date	Vital signs and laboratory findings	Treatment
1	M	57	FOLFIRI/Avastin	7	34/POD#7	BT 39.2, WBC 10k, CRP 5.26	Loop ileostomy
2	F	34	XELOX/Avastin	9	28/POD#7	Tachycardia, WBC 30k, CRP23	Antibiotics, PCD insertion
3	M	63	FOLFIRI/Avastin	5	21/POD#10	BT 38.3, WBC 22k, CRP 10	Antibiotics, PCD insertion
4	M	68	FOLFIRI/Avastin	4	47/POD#11	BT 38.3, WBC 20k, CRP 6.8	Loop ileostomy
5	M	50	FOLFIRI/Cetuximab	11	30/POD#8	Tachycardia, WBC 18k, CRP 8	Antibiotics
6	F	53	FOLFIRI/Avastin	8	44/POD#7	Septic shock, WBC 17k, CRP 11	Loop ileostomy
7	F	56	FOLFOX/Avastin	4	43/POD#23	BT 38.4, WBC 20k, CRP 37	Antibiotics
8	F	45	FOLFIRI/Avastin	15	46/POD#9	BT 39.1, tachycardia, WBC19k, CRP 9	Irrigation and drain replacement
9	M	43	FOLFIRI/Avastin	3	28/POD#27	BT38.1, normal WBC, CRP 1.9	Loop ileostomy
10	M	69	FOLFOX/Avastin	3	56/POD#6	Tachycardia, Tachypnea, WBC 8k CRP 6.9	Loop ileostomy
11	M	69	FOLFIRI/Avastin	17	34/POD#9	Septic shock, WBC 17k, CRP 2.28	Loop ileostomy
12	M	36	FOLFOX/Avastin	9	50/POD#23	V/S stable, normal WBC, CRP 2.5	Antibiotics
13	F	45	FOLFOX/Avastin	2	47/POD#13	V/S stable, normal WBC, CRP 6	Antibiotics
14	F	23	FOLFIRI/Avastin	42	28/POD#7	Sepsis, WBC 13k, CRP 13	Loop ileostomy

BT, Body temperature; WBC, White blood cell; CRP, C-reactive protein; PCD, Percutaneous catheter drainage; V/S, Vital sign.

Eight (57.1%) patients underwent emergent operations. Seven underwent intra-abdominal irrigation with loop ileostomy and 1 underwent irrigation and drainage only because this patient already had an ileostomy from the initial operation. Three (18.75%) patients were treated with antibiotics with percutaneous catheter drainage insertion for complicated fluid collection. Three (18.75%) patients were treated with antibiotics only.

Among 14 patients, 3 (21.4%) patients had an ileostomy from the primary operation. One patient underwent irrigation and drainage because she was diagnosed with sepsis with complicated fluid collection with anastomosis site dehiscence in the CT examination. One patient was treated with antibiotics with percutaneous catheter drainage insertion. He had a high fever of over 38 °C with tachycardia and bacteremia. The CT showed air containing fluid collection abutting the anastomosis site with localized peritonitis. One patient was treated with antibiotics only because his vital signs were stable and CT showed air containing fluid collection suspicious of connection with anastomosis site with localized peritonitis. All patients with a stoma underwent ileostomy take down after the end of chemotherapy treatment with the confirmation of anastomosis site healing by colon fluoroscopy using gastrografin. In patients who didn’t need ileostomy for anastomosis site leakage, median days of fistula to close was 18days (range, 12-62). In patient treated with laparotomy with ileostomy, median months of fistula to close was 5.5 months (range, 3-24).

In multivariable analysis, chemotherapy agent was independent factor associated with anastomosis site leakage (P = 0.008, **Table 4**). In the bevacizumab group, the discontinuation period before surgery was independent factor associated with anastomosis site leakage in multivariable analysis (P = 0.031, **Table 5**).

**Table 4:** Univariable and multivariable analyses of factors associated with anastomosis site leakage in stage IV colorectal cancer patients.

	Univariable analysis	Multivariable analysis		
	P-value	Odds ratio	95%CI	P-value
Age	0.085	0.958	0.915-1.004	0.071
Sex	0.852			
BMI	0.882			
Underlying DM	0.995			
ASA score	0.791			
Location	0.098	2.777	0.811-9.513	0.104
Chemotherapy agent	0.017	16.720	2.111-132.401	0.008
Route of access	0.116			
Diverting stoma	0.561			
Cancer obstruction	0.468			
Cancer perforation	0.991			
EBL	0.933			
Transfusion	0.954			
T stage	0.939			
N stage	0.947			
Harvested LN	0.855			

BMI, Body mass index; EBL, Estimated blood loss; LN, Lymph node.

**Table 5:** Univariable and multivariable analyses of factors associated with anastomosis site leakage in the bevacizumab group.

	Univariable analysis	Multivariable analysis		
	P-value	Odds ratio	95%CI	P-value
Age	0.038	0.963	0.916-1.013	0.141
Sex	0.877			
BMI	0.799			
Underlying DM	0.671			
ASA score	0.688			
Location	0.083	3.646	0.921-14.430	0.065
Route of access	0.091	0.391	0.111-1.385	0.065
Diverting stoma	0.672			
Cancer obstruction	0.472			
Cancer perforation	0.993			
EBL	0.911			
Transfusion	0.772			
T stage	0.810			
N stage	0.807			
Harvested LN	0.501			
Drug holiday	0.023	4.141	1.136-15.097	0.031
Number of doses	0.290			

BMI, Body mass index; EBL, Estimated blood loss; LN, Lymph node.

## Discussion

Monoclonal antibodies are chemotherapeutic agents targeting specific receptors on cancer cells (10). Bevacizumab, a monoclonal antibody, acts as an anti-angiogenic agent inhibiting VEGF-A (2). The addition of bevacizumab to fluorouracil-based combination therapy resulted in significant improvement in survival among patients with stage IV colorectal cancer (11, 12). However, because of the anti-angiogenic effects, many studies have reported the complications of surgical wound healing or gastrointestinal perforation in patients treated with bevacizumab (13–16). In previous studies, bevacizumab-associated GI perforation was seen in 1.5% – 1.6% of the patients with metastatic colorectal cancer (4, 11). Also bevacizumab is considered a preoperative risk factor for colorectal anastomotic leakage (17). And some studies reported spontaneous delayed anastomotic complications associated with bevacizumab (18, 19).

In our study, postoperative anastomosis site leakage was observed in 5.4% of the patients with stage IV colorectal cancer treated with preoperative chemotherapy/chemoradiotherapy. Of the anastomosis site leakage patients, 93.75% were treated with bevacizumab combined with FOLFOX/FOLFIRI/XELOX. Patients treated with bevacizumab showed significantly higher anastomosis site leakage compared to the non-bevacizumab group. Bevacizumab was also a factor associated with anastomosis site leakage in stage IV colorectal patients. Koscielny et al. reported that bevacizumab was associated with significantly higher anastomosis site leakage in the non-ileostomy group who underwent debulking surgery for ovarian cancer (20). Also, Uehara et al. reported 27.8% of anastomosis site leakage in rectal cancer patients who underwent neoadjuvant XELOX+Bevacizumab followed by total mesorectal excision (21).

Anastomotic leakage after rectal cancer surgery commonly occurs in the early postoperative period within 7 days (22, 23). Previous studies revealed that treatment with bevacizumab could be associated with delayed anastomosis site perforation, even 15 months after surgery (18, 19). In our study, the median time to leakage was 9 days (range, 6 – 27 days), which was longer than anastomotic leakage after surgery in patients without bevacizumab treatment. Therefore, even if time has passed since the operation, if the patient complains of anal pain or bleeding, anastomotic leakage should be suspected, and further examinations should be performed.

Bevacizumab has a long terminal half-life (20 days) and the bevacizumab prescribing information recommends discontinuing bevacizumab at least 4 weeks before surgery (24).

The NCCN guidelines suggest withholding bevacizumab at least 6 weeks prior to surgery (8). Yoshioka et al. reported that the interval between bevacizumab and surgery was not a risk factor for anastomotic leakage, but study was based on a median interval of 9 weeks so effect of bevacizumab on anastomosis site healing would be small (25). In this study, anastomosis site leakage was significantly higher in patients with discontinuation dates shorter than 5 weeks and there was no significant difference between the two groups when compared based on a 4 or 6-week discontinuation interval. Therefore, the discontinuation of bevacizumab is recommended at least 5 weeks prior to major surgery.

Despite the limitation that this study was a retrospective study from a single-center, to our knowledge, this was the first study to evaluate the effect of bevacizumab on anastomosis site healing in stage IV colorectal patients. Further prospective studies from multi-centers should be conducted to confirm our study results.

In conclusion, bevacizumab affected anastomosis site healing after colorectal cancer, and at least 5 weeks from bevacizumab discontinuation to surgery was associated with lower anastomosis site leakage compared to discontinuation dates shorter than 5 weeks. Thus, stage IV colorectal patients treated with bevacizumab before radical surgery for primary cancer should be carefully observed after the operation, and the period of drug discontinuation before surgery should be longer than 5 weeks to avoid anastomosis site leakage.

## Data availability statement

The raw data supporting the conclusions of this article will be made available by the authors, without undue reservation.

## Ethics statement

The studies involving human participants were reviewed and approved by the Institutional Review Board of Samsung Medical Center. Written informed consent for participation was not required for this study in accordance with the national legislation and the institutional requirements.

## Author contributions

Guarantor of integrity of the entire study: WL, YC. Study concepts and design: YC, SY. Literature research: JS, YP. Data analysis: SK, JS. Statistical analysis: SK, JH. Manuscript preparation: SK, YC, HK. Critical revision of manuscript: SK, JH, WL, SY, HK, YC, YP, JS. All authors contributed to the article and approved the submitted version.

## Acknowledgments

This research was supported by a grant of the Korea Health Technology R&D project through the Korea Health Industry Development Institute (KHIDI), funded by the Ministry of Health & Welfare, Republic of Korea (grant number: HR20C0025). This work was supported by the BK21 FOUR Project.

## Conflict of interest

The authors declare that the research was conducted in the absence of any commercial or financial relationships that could be construed as a potential conflict of interest.

## Publisher’s note

All claims expressed in this article are solely those of the authors and do not necessarily represent those of their affiliated organizations, or those of the publisher, the editors and the reviewers. Any product that may be evaluated in this article, or claim that may be made by its manufacturer, is not guaranteed or endorsed by the publisher.
